# Draft genomes of seven isolates from Danish wastewater facilities belonging to *Pseudomonas*, *Bacillus*, *Pseudochrobactrum*, *Brevundimonas*, and *Pandoraea*

**DOI:** 10.1128/MRA.00529-23

**Published:** 2023-11-15

**Authors:** Lorrie Maccario, Ana F. Silva, Joseph Nesme, Cristina I. Amador, Søren J. Sørensen, Vaughn S. Cooper, Henriette L. Røder

**Affiliations:** 1 Section of Microbiology, Department of Biology, University of Copenhagen, Copenhagen, Denmark; 2 Department of Microbiology and Molecular Genetics, University of Pittsburgh School of Medicine, Pittsburgh, Pennsylvania, USA; 3 Section of Microbiology and Fermentation, Department of Food Science, University of Copenhagen, Copenhagen, Denmark; Loyola University Chicago, Chicago, Illinois, USA

**Keywords:** biofilms, environmental microbiology, wastewater treatment

## Abstract

We report here seven draft genomes of bacterial strains from two Danish wastewater facilities, two of which might be characterized as a new group within the *Pseudomonas* and *Pseudochrobactrum* genera, respectively. These genomes will provide useful references for understanding bacterial interactions and horizontal gene transfer within bacterial communities.

## ANNOUNCEMENT

Most bacteria are found in biofilms, where they are thriving as structured and complex communities. Here, we performed genome sequencing of seven wastewater isolates to facilitate investigation of emergent interactions between bacterial species and horizontal gene transfer within bacterial biofilms.

The seven strains were isolated directly from wastewater samples taken at two facilities in Denmark, Kalundborg and Odense, on Luria broth base, Miller’s modified, with 1.5% agar plates incubated at 24°C for 96 hours. Selected isolates were restreaked three times on agar plates. DNA was extracted using DNeasyR Blood and Tissue Kit from an overnight culture of a single colony. Strains K2-sn1398, K4-sn1399, and K5-sn1400 were sequenced for short and long reads, while sp1633 to 1636 were sequenced for short reads only. For MinION Nanopore sequencing, the libraries were prepared from a new DNA extraction with Rapid Barcoding Kit SQK-RBK004 and loaded on a FLO-MIN-106 flowcell for 48 hours (Oxford Nanopore). These samples were also sequenced for short reads using Truseq libraries on NovaSeq 6000 (2 × 150 cycles; Novogene - UK). For samples with short reads only, libraries were prepared using Nextera-XT and sequenced on MiSeq at the University of Copenhagen, using Reagent Kit v3 (2 × 300 cycles; Illumina).

Quality control and adapter trimming were performed with bbduk suite v38.76 (trimq = 25) (1) and porechop v0.2.3_seqan2.1.19 (2) for short and long read sequencing, respectively. Assembly was performed using Unicycler v0.4.8 (3) with short reads only or hybrid assembly mode. Assembled contigs were smaller than 200 bp. Assembly statistics were recorded with QUAST v5.0.2 (4) and checked for completeness and contamination using CheckM lineage workflow v1.0.13 (5). Taxonomic classification was conducted with Average Nucleotide Identity (ANI) calculation against reference genomes from BV-BRC database v3.28.22 (6) using FastANI v1.32 (7). All software were used with default parameters. Annotation was performed using Prokaryotic Genome Annotation Pipeline (PGAP) (8).

*De novo* assembly and annotation resulted in seven draft genomes with a completion higher than 99% (Table 1). Taxonomic classification could be resolved at species level for five of the submitted genomes, with an ANI higher than 98%: K2-sn1398, K4-sn1399, K5-sn1400, sp1634, and sp1635 could be affiliated to *Bacillus thuringiensis*, *Pseudomonas defluvii*, *Pseudomonas brenneri, Pandoraea communis,* and *Brevundimonas diminuta*, respectively. However, two strains, sp1636 and sp1633, presented low similarity with any sequenced species. The closest relatives of *Pseudomonas* sp. sp1636 were *Pseudomonas* sp. LAM-KW06, strain CC6-YY-74, MMS21, and TM103, with an ANI of only 89.3%, 88.9%, and 88.4%, respectively, followed by representatives of *Pseudomonas lalucatii* with 85% ANI. The closest relatives of *Pseudochrobactrum* sp. sp1633 were *Pseudochrobactrum algeriensis* C130915_07 with an ANI of only 87.9%. These two strains might thus represent a new group among *Pseudomonas* and *Pseudochrobactrum* genera, respectively.

**TABLE 1:** Sequencing statistics and accession numbers

	*Bacillus thuringiensis* K2-sn1398	*Pseudomonas defluvii* K4-sn1399	*Pseudomonas brenner*i K5-sn1400	*Pseudochrobactrum* sp. sp1633	*Pandoraea communis* sp1634	*Brevundimonas diminuta* sp1635	*Pseudomonas* sp. sp1636
Isolated from	Kalundborg WWTP	Kalundborg WWTP	Kalundborg WWTP	Odense WWTP	Odense WWTP	Odense WWTP	Odense WWTP
Sequencing technology	Nanopore Minion + Illumina NovaSeq	Nanopore Minion + Illumina NovaSeq	Nanopore Minion + Illumina NovaSeq	Illumina MiSeq	Illumina MiSeq	Illumina MiSeq	Illumina MiSeq
Illumina reads no.	1,125,534	1,343,284	1,167,732	324,084	323,269	408,082	375,543
Nanopore reads no.	166,010	58,242	228,949	–	–	–	–
Nanopore reads N50	5,037	10,389	8,353	–	–	–	–
Contigs (≥200 bp) no.	16	1	1	56	126	56	58
Contigs (≥50,000 bp) no.	7	1	1	20	39	28	25
Largest contig (bp)	4,479,143	5,243,053	6,261,944	457,664	566,308	229,755	367,290
Total length (bp)	6,379,955	5,243,053	6,261,944	3,893,781	5,867,069	3,338,869	4,461,864
N50	4,479,143	5,243,053	6,261,944	265,736	114,975	95,883	191,479
GC (%)	35.45	59.71	59.78	51.59	62.34	67.04	62.86
Completeness (%)	99.32	99.59	99.18	100	99.5	99.68	99.59
Contamination (%)	0.59	0.79	1.55	0.43	0.31	0.06	0.73
Sequencing coverage (×)	296	369	255	68	36	81	61
ANI (%)	99.29	98.72	99.72	87.87	99.34	99.17	89.31
Closest relative BV-BRC accession no.	1428.1733	306.266	1284393.3	2834768.3	2508297.3	293.31	2715755.3
Biosample accession no.	SAMN33900384	SAMN33900385	SAMN33900386	SAMN33900387	SAMN33900388	SAMN33900389	SAMN33900390
Genome accession no.	JARTVI000000000	CP123067	CP122540	JARRAE000000000	JARRAD000000000	JARRAC000000000	JARRAB000000000
SRA accession no. Short reads	SRR23978664	SRR23978663	SRR23978662	SRR23978661	SRR23978660	SRR23978659	SRR23978658
SRA accession no. Long reads	SRR23978657	SRR23978656	SRR23978655	–	–	–	–
Splejsen strain collection accession no.	sn1398	sn1399	sn1400	sp1633	sp1634	sp1635	sp1636

## Data Availability

The raw data and assemblies have been deposited under the BioProject PRJNA947917 in NCBI Sequence Read Archive and Genome accession numbers listed in Table 1.The strains can be obtained from Splejsen strain collection (9) from the Section of Microbiology at the University of Copenhagen - Denmark, under the accession numbers sn1398, sn1399, sn1400, sp1633, sp1634, sp1635, and sp1636.
